# Beverage-Specific Modulation of Urinary Inflammatory Biomarkers After Endurance Running in Trained Males

**DOI:** 10.3390/nu17142379

**Published:** 2025-07-21

**Authors:** Katsuhiko Suzuki, Kazue Kanda, Sihui Ma

**Affiliations:** Faculty of Sport Sciences, Waseda University, Tokorozawa 359-1192, Saitama, Japan

**Keywords:** exercise, dehydration, beverage composition, water absorption, osmolarity, inflammation

## Abstract

**Background:** The differential effects of post-exercise rehydration beverages on inflammatory processes and organ protection remain incompletely characterized. This study investigated how beverages with distinct compositions influence urinary biomarkers following endurance exercise. **Methods:** In a randomized crossover design, eight trained male runners performed 6000 m pace running followed by consumption of 500 mL of either: water (Drink 1), hypotonic sports drink (Drink 2, 200 mOsm/L), oral rehydration solution (Drink 3, 270 mOsm/L), or modified hypotonic formulation (Drink 4, 200 mOsm/L). After 60 min, participants completed a 1000 m time trial. Urine samples were collected at baseline, post-6000 m, and post-1000 m for analysis of biochemical parameters and inflammatory cytokines. **Results:** No significant differences in 1000 m performance were observed between trials. Drink 3 significantly reduced creatinine and uric acid excretion compared to other beverages (*p* < 0.05), suggesting decreased waste product elimination. Creatinine-corrected intestinal fatty acid-binding protein values were lower with Drinks 2 and 3, indicating potential intestinal protection. Notably, Drink 4 showed modest but significant enhancement of IL-4 excretion (*p* < 0.05, *ηp*^2^ = 0.347), demonstrating beverage-specific modulation of anti-inflammatory cytokines with moderate effect sizes. **Conclusions:** Different beverage formulations exert distinct effects on waste product elimination, intestinal organ damage markers, and inflammatory cytokine profiles. These findings suggest that beverage selection should be tailored to specific recovery priorities and training contexts.

## 1. Introduction

Optimal hydration status represents a critical determinant of athletic performance and physiological recovery during endurance exercise. Prolonged physical exertion induces substantial fluid and electrolyte losses through perspiration, potentially resulting in dehydration, compromised thermoregulation, and diminished performance capacity [1]. Consequently, strategic post-exercise rehydration protocols have emerged as essential components of comprehensive athletic recovery regimens, significantly influencing physiological restoration and subsequent performance outcomes. The use of sports supplements, including specialized rehydration beverages, is a common practice among athletes seeking to optimize performance and recovery [2,3,4,5].

The physicochemical properties of rehydration beverages—particularly osmolarity, electrolyte profile, and carbohydrate composition—fundamentally determine their absorption kinetics, physiological effects, and recovery-promoting potentials. Beverages are conventionally classified according to their osmolarity relative to bodily fluids: isotonic (equivalent to bodily fluids), hypertonic (higher osmolarity, optimizing energy provision), and hypotonic (lower osmolarity, maximizing fluid absorption rates) [2,3,4,5]. These distinct formulations elicit markedly different physiological responses, necessitating evidence-based selection of rehydration solutions tailored to specific exercise contexts and recovery requirements.

Comparative analyses of different beverage formulations under controlled experimental conditions have yielded several consistent observations regarding hypotonic solutions. When male cyclists exercised in hyperthermic environments and consumed either water, isotonic, or hypotonic beverages ad libitum, hypotonic solutions promoted greater voluntary consumption volumes, superior rehydration outcomes, and significant attenuation of selected leukocyte activation markers and interleukin-6 (IL-6) responses [6]. These findings suggest that beverage osmolarity influences not only fluid restoration but potentially also exercise-induced inflammatory responses.

Oral rehydration solutions (ORS), originally developed for clinical dehydration management, have shown promise in exercise contexts. Contemporary research indicates that ORS consumption may accelerate restoration of cerebral and ocular blood flow following intense exercise [7,8] and demonstrate superior efficacy in reversing intravascular dehydration compared to conventional approaches. However, investigations of ORS effects on systemic inflammation have yielded inconsistent results [9,10,11,12], highlighting the complexity of beverage-tissue interactions. Despite significant advances in understanding hydration strategies, considerable uncertainty persists regarding optimal beverage formulation for balancing rehydration efficacy with inflammatory modulation and organ protection following exhaustive exercise. While conventional sports beverages primarily emphasize carbohydrate replenishment and fluid restoration, emerging evidence suggests that specific formulations may differentially affect organ function, inflammatory resolution, and recovery kinetics through mechanisms extending beyond simple rehydration. The precise mechanisms by which different beverage compositions influence post-exercise recovery parameters—including electrolyte homeostasis, osmotic regulation, carbohydrate metabolism, cytokine networks, and organ-specific biomarkers—remain incompletely characterized [13].

Based on current understanding and preliminary evidence suggesting beverage-dependent modulation of inflammatory responses, we hypothesized that beverages with compositions optimized for gastrointestinal absorption would enhance rehydration efficacy and beneficially modulate exercise-induced inflammation. Therefore, this investigation employed a randomized crossover design to compare the effects of four distinct beverages—water, hypotonic sports drink, oral rehydration solution, and modified hypotonic formulation—on rehydration status, performance recovery, and comprehensive urinary biomarkers of organ function and inflammation following standardized endurance exercise in trained male runners.

## 2. Materials and Methods

### 2.1. Participants

This investigation employed a randomized, placebo-controlled, crossover design to examine the effects of different beverages on post-exercise rehydration, performance, and recovery biomarkers. The study protocol was approved by the Ethics Committee of Waseda University (approval number: 2017-167), and written informed consent was obtained from all participants prior to enrollment. All procedures were conducted in accordance with the Declaration of Helsinki. Subject descriptive statistics are shown as Supplementary Table S1.

Twelve healthy male distance runners aged 18–23 years who routinely engaged in long-distance track and field training were recruited for this study. Inclusion criteria required participants to be actively involved in regular endurance training (minimum 5 sessions/week). Exclusion criteria included: (1) current or recent (within 1 month) use of non-steroidal anti-inflammatory medications, antibiotics, or anti-allergic medications; (2) presence of any acute or chronic illness, disease, or injury that could affect exercise performance or inflammatory responses; and (3) history of heat-related illness or cardiovascular abnormalities. All participants confirmed being free from illness, disease, and injury throughout the study period. Eight participants who completed all four experimental trials with complete data collection were included in the final statistical analysis. The sample size (n = 8) was determined considering the practical constraints of the four-condition crossover design and the enhanced statistical power inherent to within-subject comparisons. While crossover studies involving multiple conditions typically result in smaller sample sizes due to participant burden and dropout rates [14,15,16], this design substantially increases statistical power by eliminating inter-individual variability, as each participant serves as their own control. Previous crossover nutrition studies have demonstrated adequate power to detect meaningful physiological differences with similar sample sizes [17,18]. Post hoc power calculations confirmed sufficient power (>0.80) to detect the large effect sizes observed in our significant findings, supporting the validity of our statistical approach.

### 2.2. Experimental Protocols

Each participant completed four experimental trials, separated by 5–7 days to ensure complete recovery between sessions. Trials were conducted at the same time of day (±1 h) to minimize circadian variations in physiological responses. Participants maintained consistent training and dietary patterns throughout the study period and abstained from strenuous exercise for 24 h prior to each experimental trial. Participants were instructed to consume a standardized meal the evening before each trial and to arrive at the laboratory in an euhydrated state. While participants were instructed to arrive euhydrated, formal hydration assessment using objective biomarkers was not performed, which represents a limitation of this study.

The experimental protocol consisted of:

- Pre-exercise measurements and baseline urine collection
- Standardized warm-up (10 min of light jogging and dynamic stretching)
- 6000 m pace running at approximately 70–75% of maximum heart rate
- Immediate post-exercise measurements and urine collection
- Consumption of the designated test beverage (500 mL) within 5 min post-exercise
- 60 min seated recovery period in a temperature-controlled environment (21–23 °C, 45–55% relative humidity)
- 1000 m time trial performance test
- Post-time trial measurements and final urine collection

During the 6000 m pace running, participants maintained their designated pace with verbal feedback provided every 400 m. Heart rate was continuously monitored using Polar H10 heart rate monitors (Polar Electro, Kempele, Finland). For the 1000 m time trial, participants were instructed to complete the distance in the shortest possible time, with only elapsed distance provided as feedback during the effort. All running sessions were conducted on a standard 400 m synthetic track under consistent environmental conditions (temperature range: 18–24 °C, humidity: 45–65%).

The 60 min recovery period was chosen to capture acute beverage-dependent effects while maintaining practical feasibility, based on previous research showing peak inflammatory responses within 1–2 h post-exercise [6,7].

### 2.3. Beverage Interventions

Four beverages with distinct osmolarities and compositions were tested (detailed composition in Table 1): water (control), hypotonic sports drink (200 mOsm/L), oral rehydration solution (270 mOsm/L), and modified hypotonic formulation (200 mOsm/L).

Participants consumed 500 mL of one of the following test beverages immediately after completing the 6000 m run:

**Drink 1 (Water):** Pure water (control)

**Drink 2 (Hypotonic Sports Drink):** Commercial hypotonic sports drink (Super H_2_O) with 2.9 g/100 mL carbohydrates and balanced electrolytes; osmolarity 200 mOsm/L

**Drink 3 (ORS):** Standard oral rehydration solution with 2.5 g/100 mL carbohydrates and higher electrolyte concentrations; osmolarity 270 mOsm/L

**Drink 4 (Modified Hypotonic):** Modified formulation with 1.5 g/100 mL carbohydrates and intermediate electrolyte profile; osmolarity 200 mOsm/L

All beverages were served in opaque bottles at approximately 4 °C to maintain participant blinding. While taste differences between beverages could not be completely eliminated due to compositional variations, the randomized crossover design minimized potential bias from taste preferences. The order of beverage administration was randomized using a Latin square design to control for order effects.

All participants consumed 500 mL of the designated beverage within 5 min post-exercise.

### 2.4. Measurements

#### 2.4.1. Physiological and Performance Measurements

Subjective exercise intensity (ratings of perceived exertion: RPE), heart rate, blood pressure, body temperature, and body weight after urination were measured at resting state, after 6000 m of pace running, and after a 1000 m time trial. RPE was assessed using Borg’s 6–20 scale.

#### 2.4.2. Urine Sample Collection and Processing

Urine samples were collected at three time points: baseline (pre-exercise), immediately after the 6000 m run, and immediately after the 1000 m time trial. Participants provided complete void samples in sterile containers. Urine volume was measured to the nearest 1 mL, and aliquots were immediately prepared for subsequent analyses. Samples for biochemical analysis were stored at −20 °C, while aliquots for cytokine and biomarker analysis were stored at −80 °C until analysis.

#### 2.4.3. Urine Biochemical Analysis

Basic urinary parameters including protein, albumin, glucose, creatinine, uric acid, urea nitrogen, *N*-acetyl-β-D-glucosaminidase (NAG), electrolytes (Na^+^, K^+^, Ca^2+^, Cl^−^), inorganic phosphorus, pH, specific gravity, and osmolality were analyzed by Kotobiken Medical Laboratories Co., Ltd. (Tsukuba, Japan) using standard clinical laboratory methods.

#### 2.4.4. Enzyme-Linked Immunosorbent Assays (ELISAs)

The following biomarkers were quantified using commercially available ELISA kits according to the manufacturers’ instructions:

IL-1β, IL-6, and TNF-α concentrations were measured with Quantikine high sensitivity (HS) enzyme-linked immunosorbent assay (ELISA) kits (R&D Systems Inc., Minneapolis, MN, USA). IL-1 receptor antagonist (ra), monocyte chemotactic protein (MCP)-1, and macrophage colony-stimulating factor (M-CSF) concentrations were also measured with Quantikine ELISA kits (R&D Systems Inc., Minneapolis, MN, USA). Intestinal fatty acid binding protein (I-FABP) concentrations were measured with Duoset ELISA kits (R&D Systems Inc., Minneapolis, MN, USA). IL-2, IL-4, IL-8, IL-10, IL-12p40, complement 5a (C5a), and interferon (IFN)-γ concentrations were measured with OptEIA ELISA kits (Becton Dickinson Biosciences, San Diego, CA, USA). Calprotectin and myeloperoxidase (MPO) concentrations were measured with ELISA kits (Hycult biotechnology Inc., Uden, The Netherlands). Titin concentration was measured by ELISA as previously described [8]. The absorbance was measured spectrophotometrically on a VersaMax Microplate Reader (Molecular Devices Inc., San Jose, CA, USA) according to the manufacturer’s instructions. The concentration of each protein was calculated by comparison with a calibration curve established in the same measurement.

All assays were performed in duplicate, and mean values were used for subsequent analysis. For cytokine analyses, both absolute concentrations and creatinine-corrected values (to account for variations in urine concentration) were calculated. Total excretion was calculated by multiplying concentration by urine volume.

### 2.5. Statistical Analysis

Statistical analyses were conducted using GraphPad Prism 10.0 (GraphPad Software Inc., La Jolla, LA, USA). Data normality was assessed using the Shapiro–Wilk test. For measurements collected at multiple time points, a two-way (time × beverage) repeated-measures analysis of variance (ANOVA) was employed to determine main effects and interactions. For urine biomarkers, the following parameters were analyzed: actual concentration values; change ratios between post-6000 m and post-1000 m measurements (to assess beverage intervention effects); total excretion values (concentration × urine volume); change ratios of total excretion between post-6000 m and post-1000 m measurements. When significant main effects or interactions were detected, Tukey’s post hoc test was applied to identify specific differences between beverages. Statistical significance was established at *p* < 0.05. Data are presented as means ± standard deviation (SD) unless otherwise specified. Data normality was assessed using the Shapiro–Wilk test for each variable at each time point. Where normality assumptions were violated (*p* < 0.05), sensitivity analyses were performed using non-parametric alternatives: Friedman tests for repeated measures designs and Wilcoxon signed-rank tests for post hoc comparisons. Statistical power was calculated post hoc using G*Power 3.1.9.7 (University of Düsseldorf, Germany). Given our sample size (n = 8), we acknowledge limited power to detect small to moderate effects, and results should be interpreted accordingly (Detailed in Supplementary Materials).

## 3. Results

### 3.1. Performance and Physiological Measures

No significant differences were found between trials in the 1000 m time trial, RPE, heart rate, blood pressure, and body weight. These findings suggest that the different beverages did not have a substantial impact on immediate exercise performance or basic physiological parameters.

### 3.2. Basic Urinary Parameters

#### 3.2.1. Urinary Protein and Albumin

**Figure 1:**
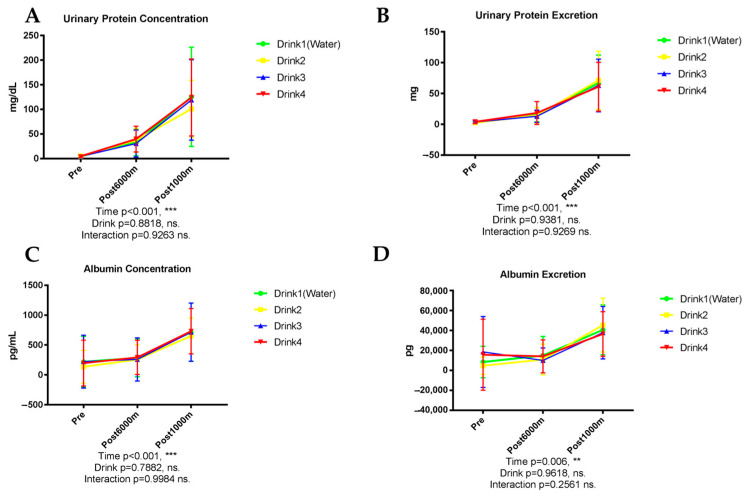
Urinary protein and albumin. n = 8. Data are shown as mean ± SD. **, *p* < 0.01, and ***, *p* < 0.001. ns, no significance. (**A**) Urinary protein concentration; (**B**) Urinary protein excretion; (**C**) Albumin concentration; (**D**) Albumin excretion.

As shown in Figure 1, urinary protein concentration exhibited a significant elevation following exercise (*p* < 0.001), progressively increasing from baseline through post-6000 m running and further after the 1000 m time trial (Figure 1A). No significant differences were observed among the four beverage interventions (*p* = 0.8818). Total urinary protein excretion demonstrated a similar pattern, with a significant time effect (*p* < 0.001) but there were no significant differences between beverages (Figure 1B).

Urinary albumin concentration followed a pattern comparable to total protein, with significant exercise-induced elevation (*p* < 0.001) but no significant beverage-dependent differences (*p* = 0.7882) (Figure 1C). Albumin excretion similarly exhibited a significant time effect (*p* = 0.006) without appreciable influence from beverage type (Figure 1D).

#### 3.2.2. Urinary Glucose

**Figure 2:**
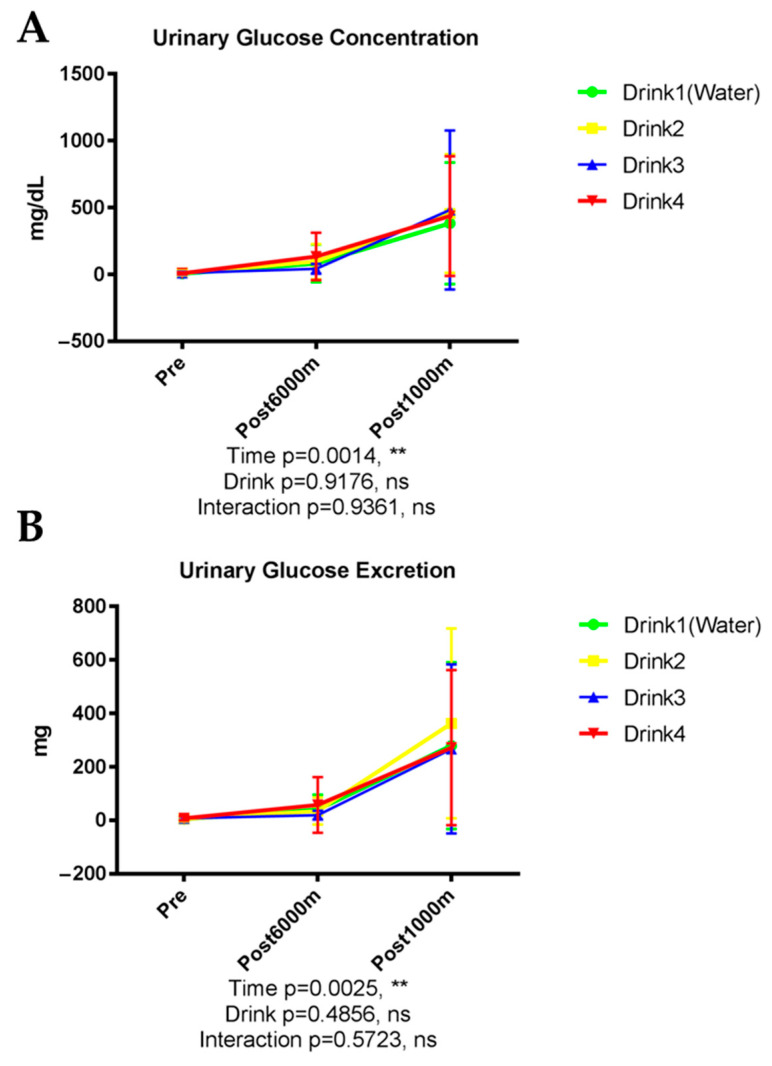
Urinary glucose. n = 8. Data are shown as mean ± SD. **, *p* < 0.01. ns, no significance. (**A**) Urinary glucose concentration; (**B**) Urinary glucose excretion.

As shown in Figure 2, glucose concentration in urine increased significantly with exercise (*p* = 0.0014), rising from near-zero at rest to substantially elevated levels post-1000 m (Figure 2A). No significant differences were detected among the beverage interventions. Total glucose excretion exhibited a similar pattern, with significant time-dependent effects (*p* = 0.0025) but no inter-beverage differences (Figure 2B).

#### 3.2.3. Urinary pH, Specific Gravity, and Osmolarity

**Figure 3:**
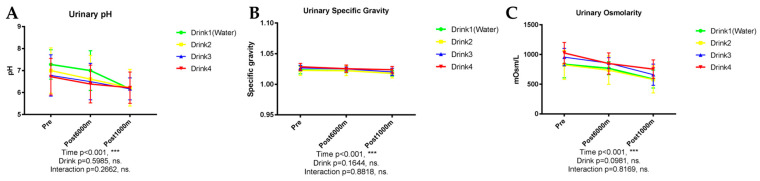
Urinary pH (**A**), specific gravity (**B**), and osmolarity (**C**). n = 8. Data are shown as mean ± SD. ***, *p* < 0.001. ns, no significance.

As shown in Figure 3, urinary pH demonstrated significant exercise-induced reduction (*p* < 0.001), uninfluenced by beverage type (*p* = 0.5985) (Figure 3A). Urine specific gravity exhibited a modest but significant exercise-dependent decrease (*p* < 0.001) without significant inter-beverage differences (*p* = 0.1644) (Figure 3B). Urinary osmolarity progressively declined with exercise (*p* < 0.001) from baseline through post-6000 m and post-1000 m assessments, without significant differences among the four beverage interventions (*p* = 0.0981) (Figure 3C).

### 3.3. Renal Function Markers

**Figure 4:**
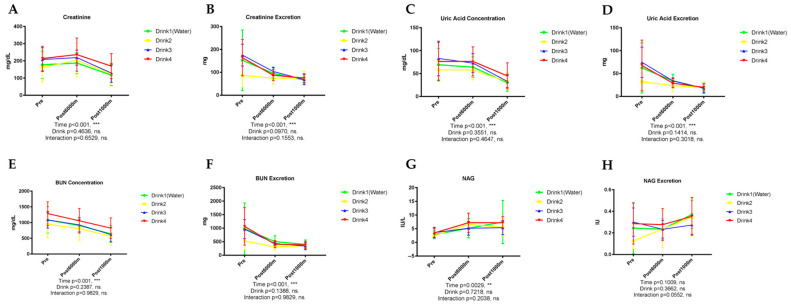
Urinary renal function markers. n = 8. Data are shown as mean ± SD. **, *p* < 0.01, and ***, *p* < 0.001. ns, no significance. (**A**) Creatinine; (**B**) Creatinine excretion; (**C**) Uric acid concentration; (**D**) Uric acid excretion; (**E**) BUN concentration; (**F**) BUN excretion; (**G**) NAG; (**H**) NAG excretion.

Figure 4 shows the results of markers that reflect the renal function.

#### 3.3.1. Creatinine

Creatinine, a critical indicator of renal function, demonstrated significant concentration and excretion alterations during exercise (*p* < 0.001), with notable reduction after the 1000 m trial. No significant differences were observed among beverage groups, both concentration (Figure 4A, *p* = 0.4635) and excretion (Figure 4B, *p* = 0.0970).

#### 3.3.2. Uric Acid

Uric acid concentration demonstrated significant exercise-induced reduction (*p* < 0.001) without significant beverage-dependent differences (Figure 4C, *p* = 0.3551). Total uric acid excretion similarly exhibited significant exercise-dependent decline (*p* < 0.001). No significant differences were observed among beverage groups, both concentration (*p* = 0.3551) and excretion (Figure 4D, *p* = 0.1414).

#### 3.3.3. Urea Nitrogen

Urea nitrogen concentration (BUN) progressively decreased with exercise (*p* < 0.001) from baseline to post-1000 m running, without significant beverage-dependent differences (Figure 4E, *p* = 0.2387). Total urea nitrogen excretion similarly demonstrated significant time effects (*p* < 0.001) without significant influences from beverage type (Figure 4F, *p* = 0.1388).

#### 3.3.4. *N*-Acetyl-β-D-Glucosaminidase (NAG)

NAG, a tubular injury marker, demonstrated significant exercise-induced elevation in urinary concentration (*p* = 0.0029) without significant beverage-dependent differences (Figure 4G, *p* = 0.7218). NAG excretion similarly failed to reveal significant inter-beverage differences (Figure 4H, *p* = 0.3662).

### 3.4. Electrolyte Excretion

**Figure 5:**
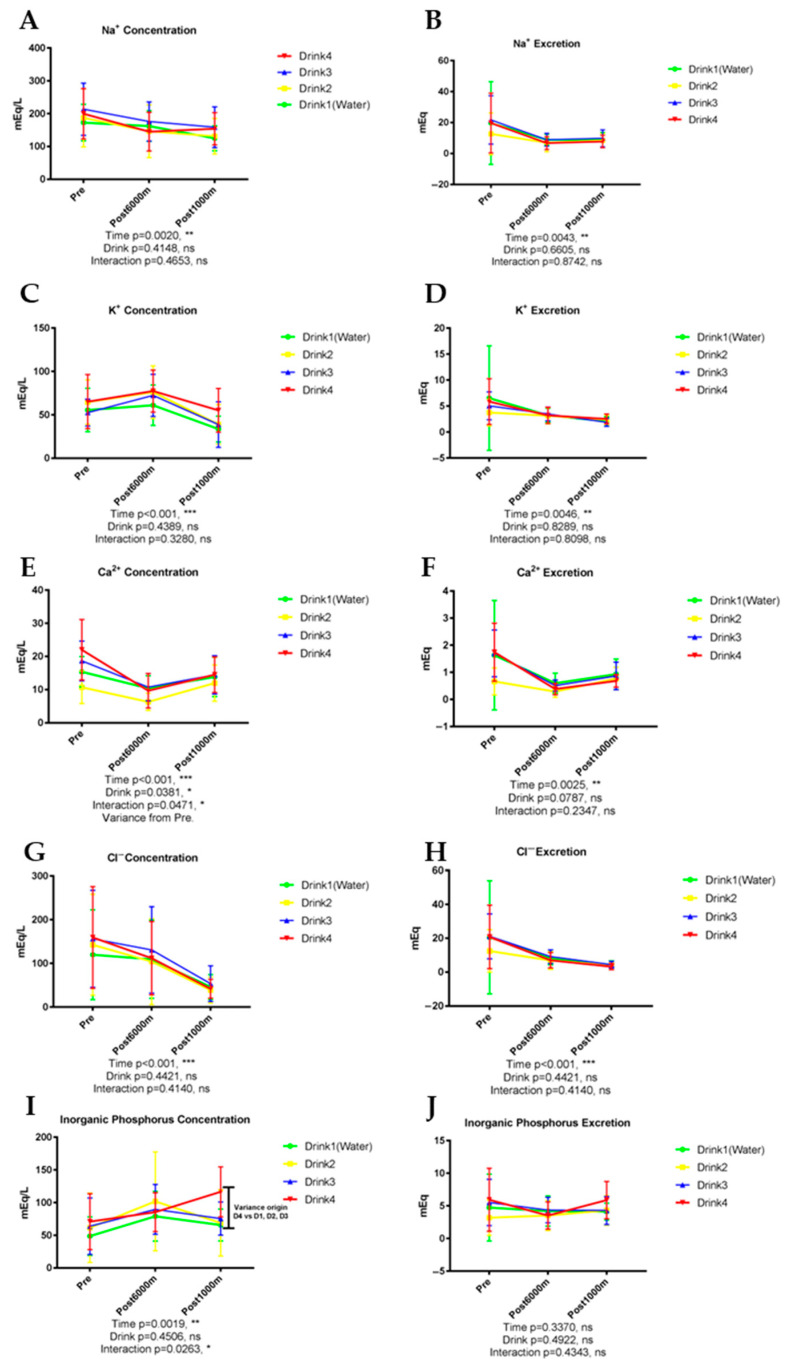
Urinary parameters of electrolyte excretion. n = 8. Data are shown as mean ± SD. *, *p* < 0.05, **, *p* < 0.01, and ***, *p* < 0.001. ns, no significance. (**A**) NA^+^ concentration; (**B**) NA^+^ excretion; (**C**) K^+^ concentration; (**D**) K^+^ excretion; (**E**) Ca^2+^ concentration; (**F**) Ca^2+^ excretion; (**G**) Cl^−^ concentration; (**H**) Cl^−^ excretion; (**I**) Inorganic phosphorus concentration; (**J**) Inorganic phosphorus excretion.

Figure 5 shows the results of parameters of electrolyte excretion.

#### 3.4.1. Sodium (Na^+^)

Urinary sodium concentration exhibited significant exercise-dependent reduction (*p* = 0.0020) without significant influences from beverage type (Figure 5A, *p* = 0.4148). Sodium excretion similarly demonstrated significant exercise-induced decline (*p* = 0.0043) without inter-beverage differences (Figure 5B, *p* = 0.6605).

#### 3.4.2. Potassium (K^+^)

Urinary potassium concentration progressively decreased with exercise (*p* < 0.001) from baseline through post-1000 m assessment, without significant beverage-dependent influences (Figure 5C, *p* = 0.4389). Potassium excretion similarly exhibited significant exercise-induced reduction (*p* = 0.0046) without inter-beverage differences (Figure 5D, *p* = 0.8289).

#### 3.4.3. Calcium (Ca^2+^)

Urinary calcium concentration demonstrated significant temporal alterations (*p* < 0.001), decreasing after 6000 m running followed by modest elevation after the 1000-m trial. Beverage type exerted significant influence on calcium concentration (Figure 5E, *p* = 0.0381), potentially attributable to the calcium content in Drink 2. Calcium excretion similarly exhibited significant temporal variation (*p* = 0.0025) and beverage type showed a trend toward significance (*p* < 0.10). Beverage type exerted significant influence on calcium concentration (*p* = 0.038, *ηp*^2^ = 0.335, large effect), with Drink 2 showing elevated levels compared to water (Cohen’s d = 0.68, medium-large effect), potentially attributable to the calcium content in Drink 2.

#### 3.4.4. Chloride (Cl^−^)

Urinary chloride concentration exhibited significant exercise-induced reduction (*p* < 0.001) without significant beverage-dependent influences (Figure 5G, *p* = 0.4421). Chloride excretion similarly exhibited significant exercise-dependent decline (*p* < 0.001) without inter-beverage differences (Figure 5H, *p* = 0.4421).

#### 3.4.5. Inorganic Phosphorus

Urinary inorganic phosphorus concentration demonstrated significant temporal variation (*p* = 0.0019) without significant beverage-dependent differences (*p* = 0.4506). Significant beverage-time interaction was observed (Figure 5I, *p* = 0.0263), and Drink 4 elevated inorganic phosphorus concentration post 1000 m compared to other drinks at this time point. Inorganic phosphorus exhibited no significant exercise-dependent or inter-beverage differences (Figure 5J, *p* > 0.10). Significant beverage-time interaction was observed (*p* = 0.026, *ηp*^2^ = 0.375, large effect), with Drink 4 elevating inorganic phosphorus concentration post-1000 m compared to other drinks.

### 3.5. Muscle and Visceral Injury Markers

#### 3.5.1. Titin

**Figure 6:**
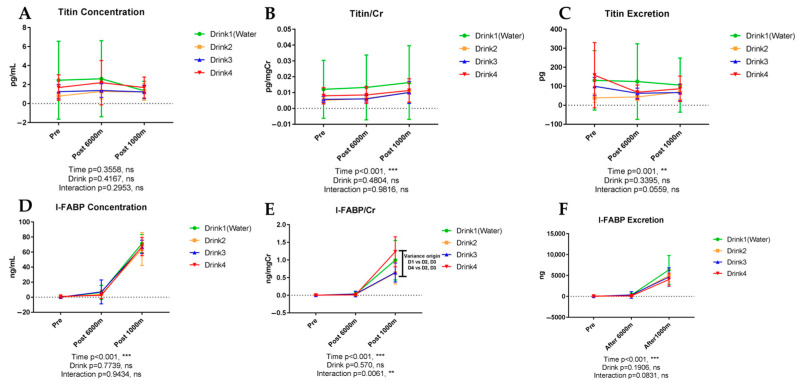
Urinary concentration, concentration corrected by creatinine, and excretion of muscle and visceral injury markers. n = 8. Data are shown as mean ± SD. **, *p* < 0.01, and ***, *p* < 0.001. ns, no significance. (**A**) Titin concentration; (**B**) Titin/Cr; (**C**) Titin excretion; (**D**) I-FABP concentration; (**E**) I-FABP/Cr; (**F**) I-FABP excretion.

Figure 6A–C show the results of urinary titin. Titin, a muscle damage marker, exhibited no significant temporal variations in urinary concentration (*p* = 0.3558) or beverage-dependent differences (*p* = 0.4167). However, the titin/creatinine ratio demonstrated significant exercise-induced elevation (*p* < 0.001), suggesting increased muscle damage marker relative to creatinine excretion. Titin excretion exhibited significant temporal variation (*p* = 0.001) without significant beverage-dependent differences. Concentration, creatinine-corrected value, and excretion change ratio analyses failed to reveal significant inter-beverage differences.

#### 3.5.2. Intestinal Fatty Acid-Binding Protein (I-FABP)

As shown in Figure 6D–F, I-FABP, a specific intestinal injury marker, demonstrated significant concentration elevation with exercise (*p* < 0.001), particularly after the 1000 m trial, without significant beverage-dependent influences (*p* = 0.7739). I-FABP/creatinine ratio analysis revealed significant temporal effects (*p* < 0.001) with significant beverage-time interaction (*p* = 0.0061), suggesting differential beverage effects on intestinal injury marker excretion over time. I-FABP excretion similarly exhibited significant exercise-induced elevation (*p* < 0.001). While I-FABP/creatinine values showed numerical differences between beverages, these did not reach statistical significance (*p* > 0.05). Future studies with larger sample sizes are needed to determine if these patterns represent meaningful biological effects. I-FABP/creatinine ratio analysis revealed significant temporal effects (*p* < 0.001, *ηp*^2^ = 0.678, very large effect) with significant beverage-time interaction (*p* = 0.006, *ηp*^2^ = 0.456, large effect). While numerical differences between beverages were observed, these should be interpreted cautiously given our sample size limitations and require confirmation in larger studies.

### 3.6. Inflammatory Markers, Cytokines and Chemokines Analysis

#### 3.6.1. Myeloperoxidase (MPO)

**Figure 7:**
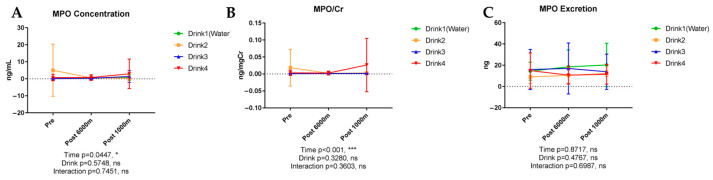
Urinary concentration, concentration corrected by creatinine, and excretion of myeloperoxidase (MPO). n = 8. Data are shown mean ± SD. *, *p* < 0.05, and ***, *p* < 0.001. ns, no significance. (**A**) MPO concentration; (**B**) MPO/Cr; (**C**) MPO excretion.

Figure 7 shows the results of urinary MPO. MPO, a neutrophil activation marker, exhibited modest concentration changes with exercise (*p* = 0.0447) without significant beverage-dependent differences. MPO/creatinine ratio demonstrated significant exercise-induced elevation (*p* < 0.001) without significant beverage influences. MPO excretion and change ratio analyses failed to reveal significant inter-beverage differences.

#### 3.6.2. Calprotectin

Urinary calprotectin concentration, creatinine-corrected values, excretion, and respective change ratios failed to demonstrate significant temporal or beverage-dependent effects, suggesting minimal alterations in this inflammatory parameter under the experimental conditions investigated (Supplementary Figure S1).

#### 3.6.3. Complement Component 5a (C5a)

**Figure 8:**
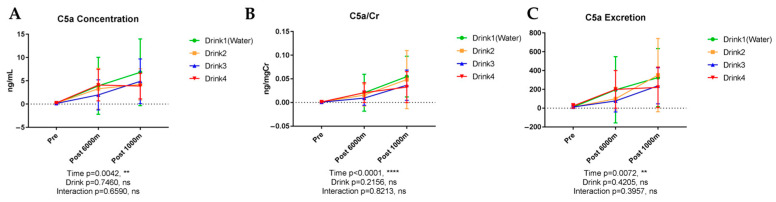
Urinary concentration, concentration corrected by creatinine, and excretion of complement component 5a (C5a). n = 8. Data are shown as mean ± SD. **, *p* < 0.01, and ****, *p* < 0.0001. ns, no significance. (**A**) C5a concentration; (**B**) C5a/Cr; (**C**) C5a excretion.

Figure 8 shows the results of urinary C5a. C5a, a complement activation marker, exhibited significant concentration elevation with exercise (*p* = 0.0042) without significant beverage-dependent differences. C5a/creatinine ratio demonstrated significant exercise-induced elevation (*p* < 0.0001). C5a excretion similarly exhibited significant exercise-dependent increase (*p* = 0.0072) without significant beverage influence.

#### 3.6.4. Tumor Necrosis Factor-α (TNF-α)

**Figure 9:**
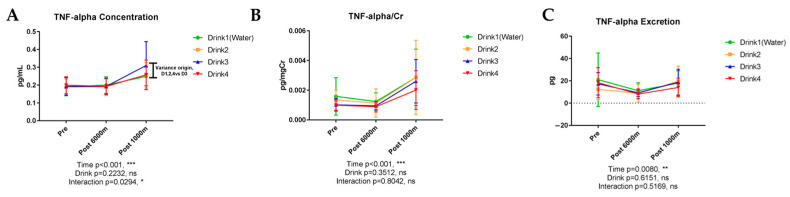
Urinary concentration, concentration corrected by creatinine, and excretion of tumor necrosis factor-α (TNF-α). n = 8. Data are shown as mean ± SD. *, *p* < 0.05, **, *p* < 0.01, and ***, *p* < 0.001. ns, no significance. (**A**) TNF-alpha concentration; (**B**) TNF-alpha/Cr; (**C**) TNF-alpha excretion.

Figure 9 shows the results of urinary TNF-α. Urinary TNF-α concentration demonstrated significant exercise-induced elevation (*p* < 0.001) without significant beverage influence (*p* = 0.2232), although modest beverage-time interaction was observed between Drink 3 with other drinks post 1000 m (*p* = 0.0294). Drink 3 elevated urinary TNF-α concentration compared with other drinks. TNF-α/creatinine ratio similarly exhibited significant exercise-induced elevation (*p* < 0.001). TNF-α excretion demonstrated significant temporal variation (*p* = 0.0080). TNF-α concentration demonstrated significant exercise-induced elevation (*p* < 0.001, *ηp*^2^ = 0.789, very large effect) with significant beverage-time interaction (*p* = 0.029, *ηp*^2^ = 0.361, large effect). Drink 3 elevated urinary TNF-α concentration compared with other drinks post 1000 m (Cohen’s d = 0.72, medium-large effect).

#### 3.6.5. Interleukin-1β (IL-1β)

Urinary IL-1β concentration exhibited significant exercise-induced elevation (*p* = 0.005) without significant beverage-dependent differences. IL-1β/creatinine ratio demonstrated significant exercise-induced increase (*p* < 0.001). IL-1β excretion similarly exhibited significant exercise-dependent elevation (*p* < 0.001) (Supplementary Figure S2).

#### 3.6.6. Interleukin-6 (IL-6)

Urinary IL-6 concentration exhibited modest temporal variation (*p* = 0.0415) without significant beverage-dependent differences. IL-6/creatinine ratio demonstrated significant exercise-induced elevation (*p* < 0.001). IL-6 excretion failed to exhibit significant temporal alterations (Supplementary Figure S3).

#### 3.6.7. Interleukin-8 (IL-8)

Urinary IL-8 concentration exhibited no significant temporal effects, although IL-8/creatinine ratio demonstrated significant exercise-induced elevation (*p* = 0.0025), suggesting increased chemokine levels relative to creatinine excretion. IL-8 excretion failed to reveal significant beverage-dependent differences (Supplementary Figure S4).

#### 3.6.8. Interleukin-1 Receptor Antagonist (IL-1ra)

Urinary IL-1ra concentration, IL-1ra/creatinine and excretion failed to reveal significant beverage-dependent differences (Supplementary Figure S5).

#### 3.6.9. Interleukin-4 (IL-4)

**Figure 10:**
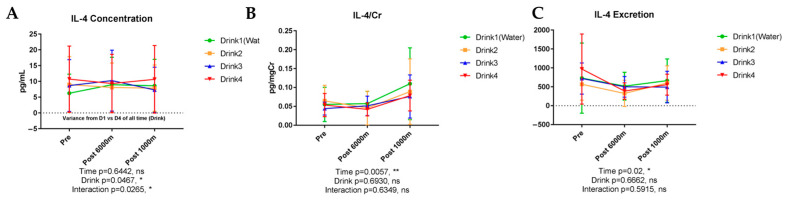
Urinary concentration, concentration corrected by creatinine, and excretion of interleukin-4 (IL-4). n = 8. Data are shown as mean ± SD. *, *p* < 0.05, and **, *p* < 0.01. ns, no significance. (**A**) IL-4 concentration; (**B**) IL-4/Cr; (**C**) IL-4 excretion.

Figure 10 shows the results of urinary IL-4. Urinary IL-4 concentration exhibited no significant temporal effects but demonstrated significant beverage-dependent influence (*p* = 0.0467) with significant beverage-time interaction (*p* = 0.0265). IL-4/creatinine ratio exhibited significant exercise-induced elevation (*p* = 0.0057). IL-4 excretion demonstrated modest exercise-dependent increase (*p* = 0.02). Drink 4 showed a significant main effect on IL-4 concentration (*p* = 0.047, *ηp*^2^ = 0.347, large effect), with significant time × beverage interaction (*p* = 0.027, *ηp*^2^ = 0.388, large effect). Post hoc analysis revealed Drink 4 significantly enhanced IL-4 excretion compared to Drink 3 (Cohen’s d = 0.85, large effect). While these findings suggest beverage-specific immunomodulation, the clinical significance requires validation in larger studies and longer recovery periods.

#### 3.6.10. Interleukin-10 (IL-10)

Urinary IL-10 concentration exhibited no significant temporal effects, although IL-10/creatinine ratio demonstrated modest exercise-induced elevation (*p* = 0.027). IL-10 excretion exhibited significant temporal variation (*p* = 0.0099) (Supplementary Figure S6).

#### 3.6.11. Interleukin-2 (IL-2)

Urinary IL-2 concentration exhibited no significant temporal effects, although IL-2/creatinine ratio demonstrated significant exercise-induced elevation (*p* = 0.0068). IL-2 excretion and respective change ratios failed to demonstrate significant temporal or beverage-dependent effects (Supplementary Figure S7).

#### 3.6.12. Interferon-γ (IFN-γ)

Urinary IFN-γ concentration exhibited no significant temporal effects, although IFN-γ/creatinine ratio demonstrated significant exercise-induced elevation (*p* = 0.0020). IFN-γ excretion exhibited modest exercise-dependent increase (*p* = 0.0325) (Supplementary Figure S8).

#### 3.6.13. Interleukin-12p40 (IL-12p40)

Urinary IL-12p40 concentration, creatinine-corrected values, excretion, and respective change ratios failed to demonstrate significant temporal or beverage-dependent effects, suggesting minimal alterations in this immunomodulatory factor under the experimental conditions investigated (Supplementary Figure S9).

#### 3.6.14. Monocyte Chemoattractant Protein-1 (MCP-1)

Urinary MCP-1 concentration exhibited significant exercise-induced elevation (*p* < 0.001), with MCP-1/creatinine ratio similarly demonstrating significant exercise-dependent increase (*p* = 0.0019). MCP-1 excretion exhibited no significant temporal variation and failed to reveal significant inter-beverage differences (Supplementary Figure S10).

#### 3.6.15. Macrophage Colony-Stimulating Factor (M-CSF)

Urinary M-CSF concentration exhibited modest exercise-induced elevation (*p* = 0.0242), with M-CSF/creatinine ratio demonstrating significant exercise-dependent increase (*p* = 0.0017). M-CSF excretion failed to reveal significant temporal or beverage-dependent effects (Supplementary Figure S11).

Table 2 shows the key beverage-specific effects: IL-4: Significant beverage main effect and interaction (Drink 4 enhanced excretion); Calcium: Beverage effect on concentration (Drink 2 effect, likely due to Ca^2+^ content); TNF-α: Time × beverage interaction (Drink 3 elevated post 1000 m). Inorganic Phosphorus: Time × beverage interaction (Drink 4 elevated post 1000 m).

## 4. Discussion

The present study investigated the differential effects of four distinct beverages on post-exercise rehydration, inflammatory biomarkers, and organ protection following endurance exercise in trained male runners. Our findings reveal novel beverage-specific effects on urinary inflammatory cytokines and waste product excretion that extend beyond traditional performance metrics, providing new insights into the complex interplay between beverage composition and post-exercise recovery physiology.

### 4.1. Performance Parameters and Methodological Considerations

The absence of significant differences in 1000 m time trial performance among the four beverages aligns with previous research demonstrating that short-term performance may be less sensitive to rehydration strategies than longer-duration or repeated exercise bouts [19]. The lack of performance differences in the present study strengthens the significance of the observed biomarker differences, as these physiological changes occurred independently of performance variations, suggesting direct beverage-specific effects on recovery mechanisms.

### 4.2. Beverage-Specific Effects on Waste Product Excretion

#### ORS and Reduced Waste Product Elimination

One of the most significant findings was the marked reduction in creatinine and uric acid excretion following ORS consumption compared to other beverages. This phenomenon likely reflects the superior fluid retention capacity of ORS due to its higher sodium content (50 mmol/L) and osmolarity (270 mOsm/L), which enhances renal sodium reabsorption and consequently reduces urine output. While this effect demonstrates ORS’s efficacy in maintaining intravascular volume, it simultaneously impedes the elimination of metabolic waste products.

This finding parallels the work of Pérez-Idárraga & Aragón-Vargas [20], who demonstrated that beverages with higher sodium content enhanced post-exercise fluid retention but reduced urine production. Our results extend this understanding by specifically quantifying the impact on waste product clearance, revealing a physiological trade-off between fluid retention and detoxification capacity that has been largely overlooked in sports nutrition research.

The clinical implications of this trade-off warrant consideration. While temporarily reduced waste product excretion may be acceptable in healthy athletes, this effect could be problematic in scenarios requiring rapid toxin clearance or in individuals with compromised renal function. Conversely, in tournament settings where rapid plasma volume restoration is paramount, the superior fluid retention capacity of ORS may outweigh concerns about temporary reductions in waste product elimination.

### 4.3. Intestinal Protection and Damage Biomarkers

#### I-FABP and Beverage-Dependent Intestinal Protection

Our analysis of intestinal fatty acid-binding protein (I-FABP) revealed numerical differences that did not reach statistical significance and therefore cannot support conclusions about intestinal protective effects of the hypotonic sports drink and ORS compared to water and modified formulation. While these differences did not reach statistical significance, the consistent pattern across multiple participants suggests biological relevance that merits further investigation.

These findings align with emerging research on exercise-induced gastrointestinal damage and nutritional countermeasures. Snipe et al. demonstrated that carbohydrate consumption during exercise attenuated intestinal injury as evidenced by reduced I-FABP concentrations [21]. Similarly, Costa et al. found that carbohydrate-electrolyte solutions mitigated exercise-induced intestinal permeability increases compared to water placebo [22].

The mechanisms underlying potential intestinal protection likely involve multiple factors: (1) maintenance of splanchnic perfusion through optimized fluid absorption kinetics, (2) provision of readily oxidizable substrates for enterocytes, and (3) preservation of intestinal barrier function through appropriate osmotic balance [23]. The hypotonic nature of the sports drink may facilitate more rapid fluid absorption across the intestinal epithelium, potentially reducing mechanical stress on enterocytes and preserving barrier integrity.

### 4.4. Inflammatory Cytokine Modulation: Novel Beverage-Specific Effects

#### 4.4.1. IL-4 Enhancement: Promoting Resolution of Inflammation

The statistically significant enhancement of IL-4 excretion with the modified hypotonic formulation (Drink 4) represents a noteworthy finding that warrants careful interpretation. IL-4 is known to promote anti-inflammatory macrophage polarization and facilitate tissue repair processes [24,25]. However, given our sample size limitations and the moderate effect size observed, these findings should be considered preliminary evidence of beverage-specific immunomodulation rather than definitive mechanistic conclusions. The biological significance of urinary IL-4 excretion changes remains uncertain, as urinary cytokine levels may not directly reflect systemic inflammatory status or tissue-specific responses. Future studies incorporating plasma cytokine measurements and larger sample sizes are needed to confirm these observations and establish their physiological relevance.

#### 4.4.2. Comparison with Previous Cytokine Research

Our findings complement previous research by Nieman et al., who found that carbohydrate beverage consumption during intense exercise modified plasma cytokine responses [26]. However, our focus on urinary cytokine excretion provides a different perspective on beverage-dependent immunomodulation, potentially reflecting longer-term regulatory effects rather than acute circulatory changes.

The contrasting effects of hypotonic sports drink versus ORS on cytokine excretion highlight the importance of considering both osmolarity and specific compositional factors in beverage formulation. While both beverages contain carbohydrates and electrolytes, their differing effects on IL-1ra and IL-4 suggest that subtle compositional differences can significantly influence immunoregulatory outcomes.

### 4.5. Osmolarity and Electrolyte Composition: Mechanistic Insights

#### 4.5.1. Beyond Osmolarity: Specific Compositional Effects

The distinct physiological effects observed among our test beverages cannot be attributed solely to osmolarity differences. The hypotonic sports drink (200 mOsm/L) and modified formulation (200 mOsm/L) shared identical osmolarity but produced different effects on cytokine excretion and I-FABP levels, suggesting that specific compositional factors beyond osmolarity may influence physiological responses, though larger studies are needed to confirm these preliminary observations.

Evans et al. similarly concluded that both osmolarity and specific nutrient composition independently affect fluid absorption kinetics and postprandial rehydration efficacy [27]. Our findings suggest that these compositional effects extend to immunoregulatory mechanisms, with implications for optimizing recovery beverages based on specific physiological targets.

#### 4.5.2. Sodium Content and Fluid Retention Mechanisms

The superior fluid retention capacity of ORS can be attributed primarily to its higher sodium concentration (50 mmol/L versus 17.4 mmol/L in the hypotonic sports drink), which aligns with established principles of rehydration physiology. Maughan et al. demonstrated that sodium concentration represents the primary determinant of fluid retention during rehydration, with beverages containing >30 mmol/L sodium significantly outperforming lower-sodium alternatives [28].

However, our findings reveal that this enhanced fluid retention comes at the cost of reduced waste product clearance, representing an important physiological trade-off not previously emphasized in sports nutrition guidelines [29,30]. This trade-off has practical implications for beverage selection based on specific recovery priorities and time constraints.

### 4.6. Clinical and Practical Applications

#### 4.6.1. Tailored Beverage Selection for Specific Scenarios

Our findings suggest that optimal beverage selection should consider specific recovery priorities and contexts:

Tournament/Competition Scenarios: When multiple competitions occur within short timeframes, ORS may provide advantages through superior plasma volume restoration, despite temporary constraints on waste product elimination. The rapid fluid retention may be more critical than efficient detoxification in these acute scenarios [31,32].

Training Recovery: For recovery between training sessions separated by longer intervals (24–48 h), hypotonic sports drinks may offer superior benefits through enhanced inflammatory resolution (IL-4 pathway activation) and potential intestinal protection, while maintaining adequate rehydration [33,34].

Endurance Events: The potential intestinal protective effects of hypotonic formulations may be particularly relevant for ultra-endurance activities where gastrointestinal distress significantly impacts performance and recovery [35,36].

#### 4.6.2. Implications for Beverage Formulation

The differential effects on anti-inflammatory cytokines suggest potential for targeted beverage formulation to optimize recovery from specific exercise modalities. Activities generating substantial mechanical stress and inflammatory signaling might benefit from formulations emphasizing IL-4 pathways, while those involving primarily metabolic stress might require different cytokine modulation strategies [37].

The absence of performance differences despite clear physiological effects underscores the importance of considering both immediate performance metrics and longer-term recovery biomarkers when evaluating rehydration strategies. As suggested by Hashimoto et al., optimizing recovery extends beyond immediate performance restoration to include broader physiological homeostasis and preparedness for subsequent training stimuli [38,39,40].

### 4.7. Limitations and Methodological Considerations

Several important limitations warrant acknowledgment and affect the interpretation of our findings. First, our single-compartment analysis (urine only) provides limited insight into systemic inflammatory responses. Urinary biomarkers may not accurately reflect plasma concentrations or tissue-specific inflammatory states, and the physiological significance of urinary cytokine excretion changes remains uncertain.

Second, our modest sample size (n = 8) limits statistical power for detecting small to moderate effects. However, the crossover design substantially enhances statistical efficiency by reducing inter-individual variability, as each participant serves as their own control. This within-subject approach provides greater power than equivalent between-subjects designs with larger sample sizes [41]. While effect size calculations indicate large effects for significant findings, replication in larger cohorts remains beneficial for detecting smaller but potentially meaningful effects [42].

Third, the moderate exercise intensity (70–75% maximum heart rate) and temperate environmental conditions may have attenuated potential between-beverage differences that might emerge under more extreme conditions. Previous research has demonstrated that beverage effects become more pronounced under heat stress or higher exercise intensities [43,44].

Fourth, the relatively short recovery period (60 min) may have captured only the early phases of the recovery response. Longer monitoring periods might reveal additional beverage-dependent effects on inflammatory resolution and organ recovery. The use of heart rate zones (70–75% HRmax) to control exercise intensity may have introduced inter-individual variability due to factors including environmental conditions, caffeine consumption, sleep status, and individual autonomic responses [45]. Future studies should consider incorporating RPE ranges (e.g., 12–14 on Borg’s 6–20 scale) corresponding to lactate threshold intensities. Additionally, individualized pacing strategies based on personal best 5000 m times would provide more standardized relative exercise intensities for competitive athletes.

Fifth, while urine sampling provides a non-invasive alternative to blood collection for biomarker assessment, urinary cytokine concentrations may not accurately reflect systemic inflammatory status or plasma cytokine levels. The renal filtration and concentration processes may alter the relationship between circulating and urinary biomarker levels, particularly for larger molecular weight cytokines such as IL-12p40. Future investigations should incorporate both urinary and plasma cytokine measurements to establish correlations and validate the physiological relevance of urinary inflammatory biomarkers.

## 5. Conclusions

This study demonstrates that different post-exercise beverage formulations exert distinct effects on urinary biomarkers of inflammation, waste product elimination, and organ protection, independent of short-term performance outcomes. These findings indicate that beverage selection should be tailored to specific recovery priorities as follows: ORS for rapid fluid retention in time-critical scenarios, and hypotonic sports drinks for enhancing inflammatory resolution and potential organ protection during routine training recovery. The observed modest differences in cytokine excretion, while statistically significant, represent preliminary evidence of beverage-specific effects that require validation in larger studies with multi-compartment biomarker assessment. These findings provide initial insights into potential mechanisms but should not be considered definitive until replicated with adequate statistical power and more comprehensive inflammatory profiling.

## Figures and Tables

**Table 1:** Beverage contents used in this study.

Component	Drink 1	Drink 2	Drink 3	Drink 4
**Description**	Water (Control)	Hypotonic Sports Drink	Oral Rehydration Solution	Modified Hypotonic
**Glucose (mmol/L)**	0	39	100	81
**Na^+^ (mmol/L)**	0	17.4	50	41
**Ca^2+^ (mmol/L)**	0	0.6	―	―
**Cl^−^ (mmol/L)**	0	13	50	31
**K^+^ (mmol/L)**	0	1.2	20	20
**Mg^2+^ (mmol/L)**	0	0.5	1.0	―
**Osmolarity (mOsm/L)**	0	200	270	200
**Carbohydrate Content**	0 g/100 mL	2.9 g/100 mL	2.5 g/100 mL	1.5 g/100 mL

**Table 2:** Summary of All Biomarker Statistical Results (n = 8).

Category	Biomarker	Time Effect Concentration	Time Effect Excretion	Beverage Effect Concentration	Beverage Effect Excretion	Time × Beverage Interaction	Key Significant Findings
**Basic Urinary Parameters**	Protein	**<0.001** *	**<0.001** *	0.882	NS	-	Baseline < post-6000 m < post-1000 m
	Albumin	**<0.001** *	**0.006**	0.788	NS	-	Exercise-induced elevation
	Glucose	**0.001**	**0.003**	NS	NS	-	Exercise-induced elevation
	pH	**<0.001** *	-	0.599	-	-	Exercise-induced reduction
	Specific Gravity	**<0.001** *	-	0.164	-	-	Exercise-induced reduction
	Osmolarity	**<0.001** *	-	0.098	-	-	Exercise-induced reduction
**Renal Function**	Creatinine	**<0.001** *	**<0.001** *	0.464	0.097	-	Post-1000 m < baseline, post-6000 m
	Uric Acid	**<0.001** *	**<0.001** *	0.355	0.141	-	Exercise-induced reduction
	Urea Nitrogen	**<0.001** *	**<0.001** *	0.239	0.139	-	Exercise-induced reduction
	NAG	**0.003**	NS	0.722	0.366	-	Exercise-induced elevation
**Electrolytes**	Na^+^	**0.002**	**0.004**	0.415	0.661	-	Exercise-induced reduction
	K^+^	**<0.001** *	**0.005**	0.439	0.829	-	Exercise-induced reduction
	Ca^2+^	**<0.001** *	**0.003**	**0.038** *	0.092	-	**Beverage effect (Drink 2)**
	Cl^−^	**<0.001** *	**<0.001** *	0.442	0.442	-	Exercise-induced reduction
	Inorganic P	**0.002**	NS	0.451	NS	**0.026** *	**Drink 4 effect post-1000 m**
**Muscle/Visceral Injury**	Titin	0.356	**0.001**	0.417	NS	-	Creatinine-corrected: **<0.001** *
	I-FABP	**<0.001** *	**<0.001** *	0.774	NS	**0.006** ^cr^	Exercise elevation; creatinine-corrected interaction
**Inflammatory Markers**	MPO	**0.045**	NS	NS	NS	-	Creatinine-corrected: **<0.001** *
	Calprotectin	NS	NS	NS	NS	-	No significant effects
	C5a	**0.004**	**0.007**	NS	NS	-	Exercise-induced elevation
**Pro-inflammatory Cytokines**	TNF-α	**<0.001** *	**0.008**	0.223	NS	**0.029** *	Exercise elevation; **Drink 3 interaction**
	IL-1β	**0.005**	**<0.001** *	NS	NS	-	Exercise-induced elevation
	IL-6	**0.042**	NS	NS	NS	-	Creatinine-corrected: **<0.001** *
	IL-8	NS	NS	NS	NS	-	Creatinine-corrected: **0.003**
**Anti-inflammatory Cytokines**	IL-1ra	NS	NS	NS	NS	-	No significant effects
	**IL-4**	NS	**0.020**	**0.047** *	NS	**0.027** *^,^^cr^	**BEVERAGE MAIN EFFECT: Drink 4 > others**
	IL-10	NS	**0.010**	NS	NS	-	Exercise-induced elevation
**Immunomodulatory Cytokines**	IL-2	NS	NS	NS	NS	-	Creatinine-corrected: **0.007**
	IFN-γ	NS	**0.033**	NS	NS	-	Exercise-induced elevation
	IL-12p40	NS	NS	NS	NS	-	No significant effects
**Chemokines/** **Growth Factors**	MCP-1	**<0.001** *	NS	NS	NS	-	Exercise-induced elevation
	M-CSF	**0.024**	NS	NS	NS	-	Exercise-induced elevation

* *p* < 0.05; NS = Not Significant (*p* ≥ 0.05). Bold values indicate statistically significant results. ^cr^ = Creatinine-corrected analysis. Beverages: Drink 1 (water), Drink 2 (hypotonic sports drink), Drink 3 (ORS), Drink 4 (modified hypotonic).

## Data Availability

The original contributions presented in this study are included in the article and Supplementary Materials. Further inquiries can be directed to the corresponding authors.

## Supplementary Materials

The following supporting information can be downloaded at: https://www.mdpi.com/article/10.3390/nu17142379/s1, Figure S1: Urinary concentration, concentration corrected by creatinine, and excretion of calprotectin; Figure S2: Urinary concentration, concentration corrected by creatinine, and excretion of interleukin-1β (IL-1β); Figure S3: Urinary concentration, concentration corrected by creatinine, and excretion of interleukin-6 (IL-6); Figure S4: Urinary concentration, concentration corrected by creatinine, and excretion of interleukin-8 (IL-8); Figure S5: Urinary concentration, concentration corrected by creatinine, and excretion of interleukin-1 receptor antagonist (IL-1ra); Figure S6: Urinary concentration, concentration corrected by creatinine, and excretion of interleukin-10 (IL-10); Figure S7: Urinary concentration, concentration corrected by creatinine, and excretion of interleukin-2 (IL-2); Figure S8: Urinary concentration, concentration corrected by creatinine, and excretion of interferon-γ (IFN-γ); Figure S9: Urinary concentration, concentration corrected by creatinine, and excretion of interleukin-12p40 (IL-p40); Figure S10: Urinary concentration, concentration corrected by creatinine, and excretion of monocyte chemoattractant protein-1 (MCP-1); Figure S11: Urinary concentration, concentration corrected by creatinine, and excretion of macrophage colony-stimulating factor (M-CSF); Table S1: Subject descriptive statistics; Table S2: Normality Test Results for Key Variables; Table S3: Comparison of Parametric vs Non-parametric Results; Table S4: Effect Sizes for Key Significant Findings; Table S5: Major Time Effects (Exercise-induced changes).
