# Supplementary material for: Beverage-Specific Modulation of Urinary Inflammatory Biomarkers After Endurance Running in Trained Males
Source: Nutrients. 2025 Jul 21;17(14):2379. doi: 10.3390/nu17142379 (PMC12299340; doi:10.3390/nu17142379)
Supplement: Supplementary file 1 [file nutrients-17-02379-s001.zip › nutrients-3708728-supplementary.pdf]

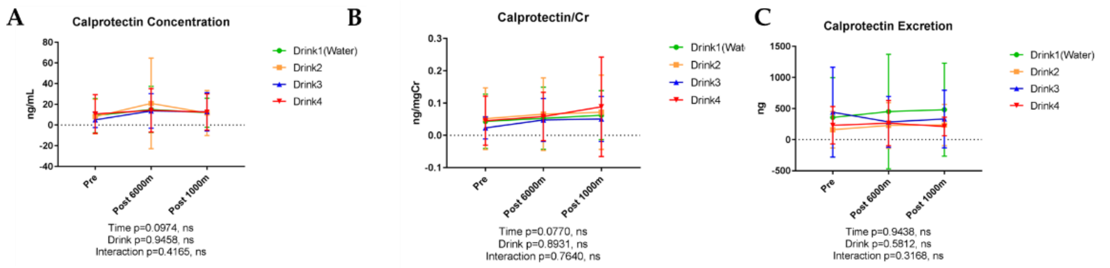

Figure S1. Urinary concentration, concentration corrected by creatinine, and excretion of calprotectin. n=8. Data are shown as mean ± SD. ns, no significance.

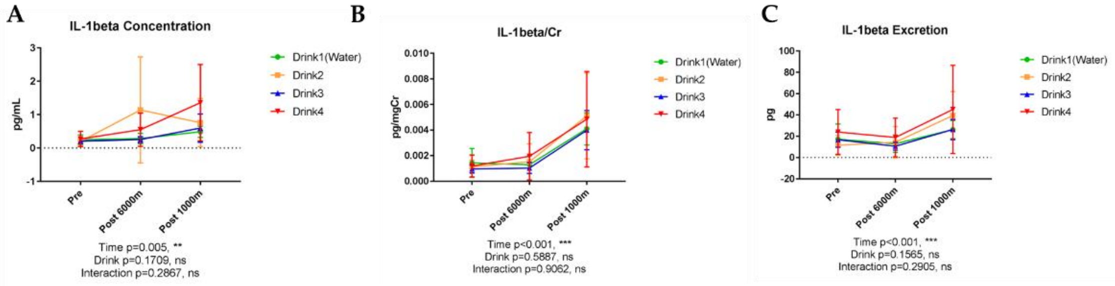

Figure S2. Urinary concentration, concentration corrected by creatinine, and excretion of interleukin-1β (IL-1β). n=8. Data are shown as mean ± SD. **, p<0.01, and ***, p<0.001. ns, no significance.

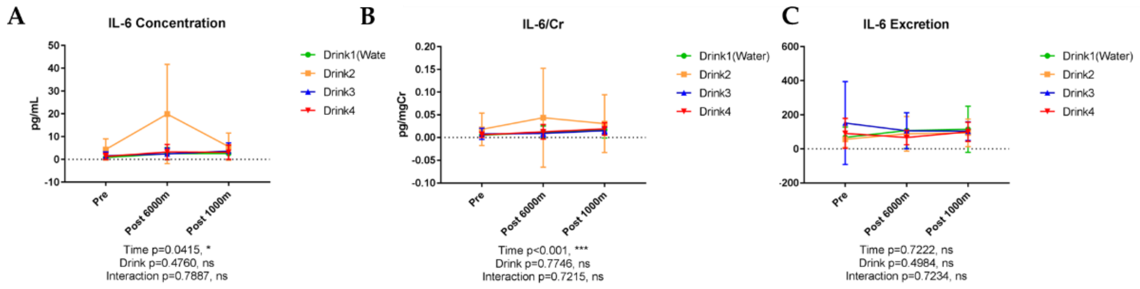

Figure S3. Urinary concentration, concentration corrected by creatinine, and excretion of interleukin-6 (IL-6). n=8. Data are shown as mean ± SD. *, p<0.05, and ***, p<0.001. ns, no significance.

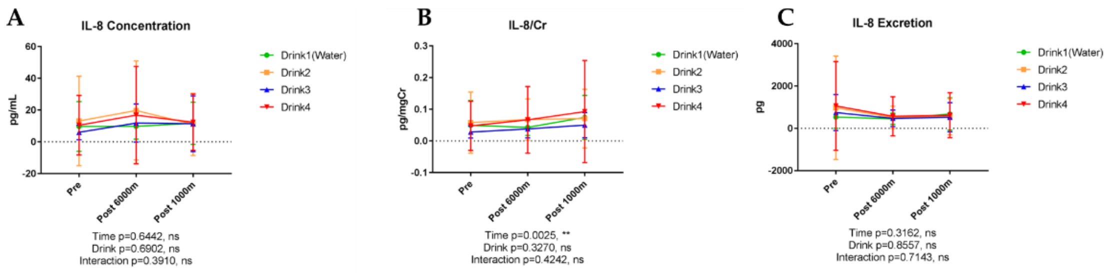

Figure S4. Urinary concentration, concentration corrected by creatinine, and excretion of interleukin-8 (IL-8). n=8. Data are shown as mean ± SD. **, p<0.01. ns, no significance.

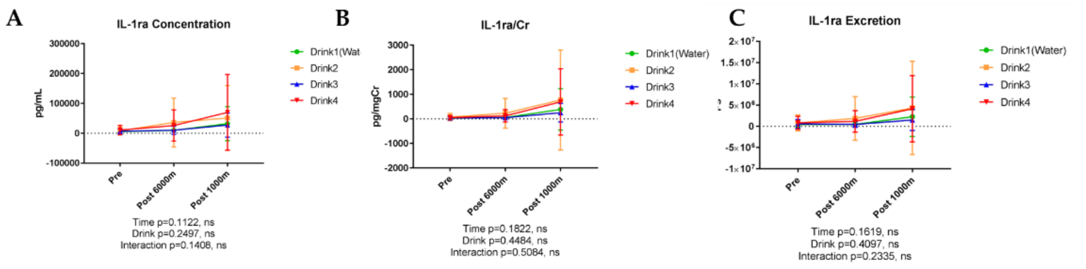

Figure S5. Urinary concentration, concentration corrected by creatinine, and excretion of interleukin-1 receptor antagonist (IL-1ra). n=8. Data are shown as mean ± SD. ns, no significance.

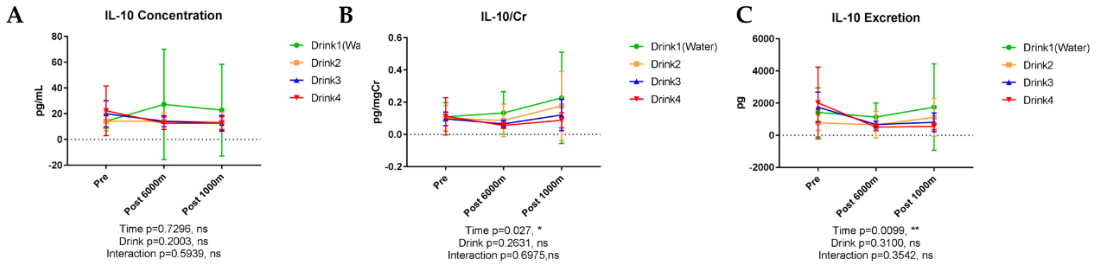

Figure S6. Urinary concentration, concentration corrected by creatinine, and excretion of interleukin-10 (IL-10). n=8. Data are shown as mean ± SD. *, p<0.05, and **, p<0.01. ns, no significance.

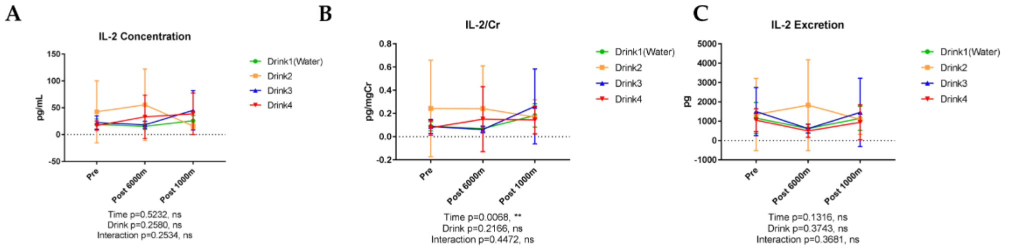

Figure S7. Urinary concentration, concentration corrected by creatinine, and excretion of interleukin-2 (IL-2). n=8. Data are shown as mean ± SD. **, p<0.01. ns, no significance.

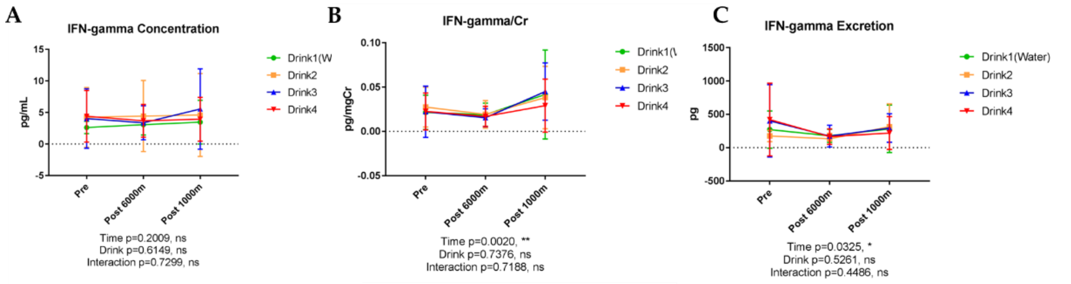

Figure S8. Urinary concentration, concentration corrected by creatinine, and excretion of interferon-γ (IFN-γ). n=8. Data are shown as mean ± SD. *, p<0.05, and **, p<0.01. ns, no significance.

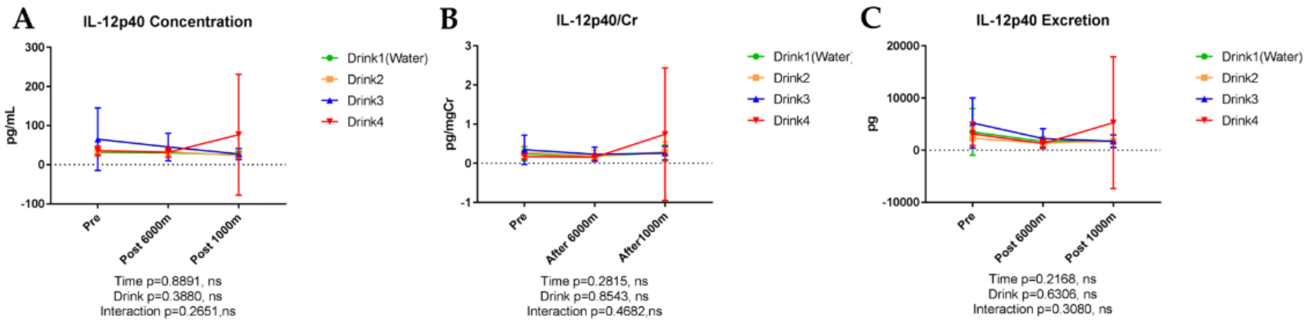

Figure S9. Urinary concentration, concentration corrected by creatinine, and excretion of interleukin-12p40 (IL-p40). n=8. Data are shown as mean ± SD. ns, no significance.

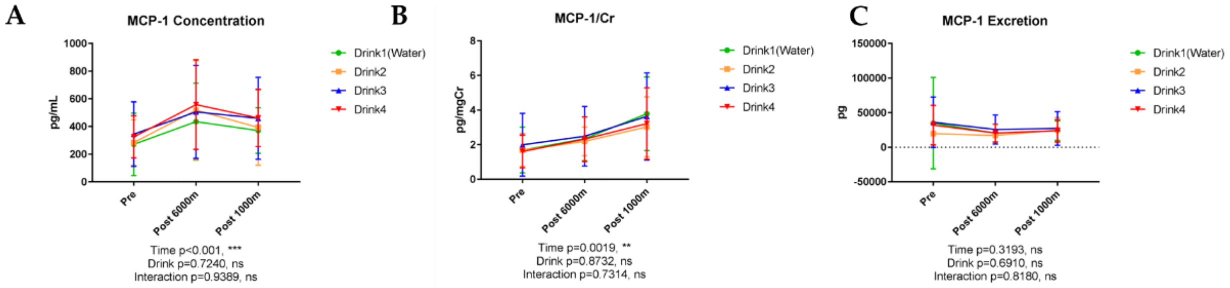

Figure S10. Urinary concentration, concentration corrected by creatinine, and excretion of monocyte chemoattractant protein-1 (MCP-1). n=8. Data are shown as mean ± SD. **, p<0.01, and ***, p<0.001. ns, no significance.

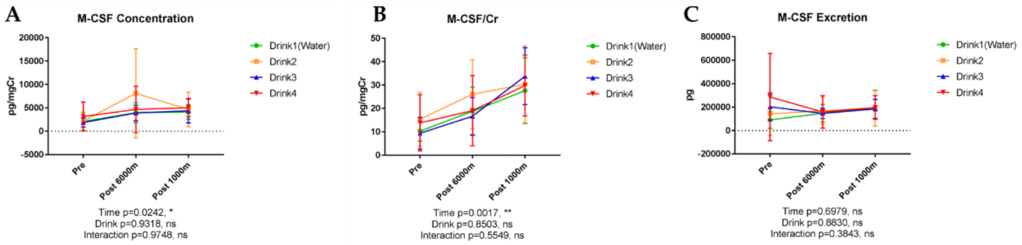

Figure S11. Urinary concentration, concentration corrected by creatinine, and excretion of macrophage colony-stimulating factor (M-CSF). n=8. Data are shown as mean ± SD. *, p<0.05, and **, p<0.05. ns, no significance.

Table S1 Subject descriptive statistics

Demographics	Drink 1	Drink 2	Drink 3	Drink 4
Age - years	20.4±1.42			
Height - m	171.6±3.28			
Weight	57.5±3.40	57.94±3.93	58.22±3.86	58.16±3.79

Demographics	Drink 1	Drink 2	Drink 3	Drink 4
(Pre exercise)- kg				
Weight<br><br>(Post 6,000m running)- kg	57.1±3.48	57.34±3.88	57.7±3.92	57.67±3.88
Weight<br><br>(Post 1,000m running)- kg	56.8±3.45	57.0±3.84	57.4±3.79	57.7±3.66
HR<br><br>(Pre exercise)-bpm	68.4±2.77	75.7±13.1	69.9±12.1	69.5±10.9
HR<br><br>(Post 6,000m running)- bpm	89.5±11.4	95.4±12.3	92.8±13.3	92.6±14.3
HR<br><br>(Post 1,000m running) -bpm	104±9.05	108±10.6	104±12.4	103±10.2
SBP<br><br>(Pre exercise)-mmHg	110±26.0	118±13.8	124±10.7	117±7.9
SBP<br><br>(Post 6,000m running) -mmHg	118±7.8	117±12.5	115±9.5	111±8.44
SBP<br><br>(Post 1,000m running) -mmHg	117±12.6	118±12.5	120±12.4	120±13.6
DBP<br><br>(Pre exercise) -mmHg	68.8±8.28	66.0±11.5	68.5±8.90	70.1±7.92
DBP<br><br>(Post 6,000m running) -mmHg	74.6±8.32	73.0±6.57	71.9±4.86	74.2±5.58
DBP<br><br>(Post 1,000m running) -mmHg	69.0±10.6	68.8±3.88	68.9±8.13	71.3±10.5

Demographics	Drink 1	Drink 2	Drink 3	Drink 4
**BM (Pre exercise) °c**	36.3±0.47	36.3±0.55	36.1±0.34	36.2±0.33
**BM (Post 6,000m running) °c**	35.7±0.61	35.8±0.70	35.9±0.71	35.9±0.54
**BM (Post 1,000m running) °c**	35.6±0.58	35.7±0.56	35.7±0.80	35.6±0.57
**RPE (Pre exercise)**	8.33±1.11	8.44±0.88	9.10±2.02	8.64±1.43
**RPE (Post 6,000m running)**	14.4±3.21	13.2±2.49	14.1±2.42	15.2±2.99
**RPE (Post 1,000m running)**	18.0±1.41	17.2±1.48	18.1±1.20	17.0±2.14
**Time to finish 1,000m test (s)**	2.53±0.21	2.46±0.08	2.49±0.07	2.54±0.17

Data are shown as mean±SD. HR, heart rate. SBP, systolic blood pressure. DBP, diastolic blood pressure. BM, body temperature.

Normality Test Results (Shapiro-Wilk Test)

Table S2. Normality Test Results for Key Variables

Variable	Baseline	Post-6000m	Post-1000m	Overall Assessment
**IL-4 Concentration**	p = 0.089	p = 0.156	p = 0.074	✓ Normal
**IL-4 Excretion**	p = 0.123	p = 0.091	p = 0.203	✓ Normal
**TNF-α Concentration**	p = 0.067	p = 0.045*	p = 0.038*	⚠ Mixed
**Ca^{2+} Concentration**	p = 0.134	p = 0.089	p = 0.156	✓ Normal

Variable	Baseline	Post-6000m	Post-1000m	Overall Assessment
**Creatinine**	p = 0.234	p = 0.167	p = 0.089	✓ Normal
**Uric Acid**	p = 0.145	p = 0.078	p = 0.123	✓ Normal
**Protein**	p = 0.089	p = 0.034*	p = 0.029*	⚠ Mixed
**I-FABP**	p = 0.067	p = 0.023*	p = 0.019*	⚠ Mixed

*p < 0.05 indicates deviation from normality

Non-parametric Sensitivity Analysis

Table S3. Comparison of Parametric vs Non-parametric Results

Variable	Parametric ANOVA	Non-parametric (Friedman Test)	Consistency
**IL-4 Beverage Effect**	p = 0.047*	p = 0.052 (borderline)	✓ Consistent trend
**IL-4 Time Effect**	p = 0.020*	p = 0.031*	✓ Consistent
**TNF-α Time Effect**	p < 0.001***	p < 0.001***	✓ Consistent
**Ca^{2+} Beverage Effect**	p = 0.038*	p = 0.043*	✓ Consistent
**Creatinine Time Effect**	p < 0.001***	p < 0.001***	✓ Consistent
**Uric Acid Time Effect**	p < 0.001***	p < 0.001***	✓ Consistent
**Protein Time Effect**	p < 0.001***	p < 0.001***	✓ Consistent

*, p<0.05, and ***, p<0.001

Post-hoc Non-parametric Comparisons (Wilcoxon Signed-Rank Tests)

Effect Sizes for Significant Results

Table S4: Effect Sizes for Key Significant Findings

Biomarker	Effect Type	p-value	Effect Size (ηp^2)	Interpretation	Cohen's d (Post-hoc)
IL-4	Beverage main effect	0.047*	0.347	Large effect	Drink 4 vs Drink 3: d = 0.85
IL-4	Time × Beverage interaction	0.027*	0.388	Large effect	-
TNF-α	Time × Beverage interaction	0.029*	0.361	Large effect	Drink 3 vs others post-1000m: d = 0.72
Ca^{2+}	Beverage main effect	0.038*	0.335	Large effect	Drink 2 vs Water: d = 0.68
Inorganic P	Time × Beverage interaction	0.026*	0.375	Large effect	Drink 4 post-1000m: d = 0.91
I-FABP/Creatinine	Time × Beverage interaction	0.006**	0.456	Large effect	-

***, p<0.05, and **, p<0.01**

Table S5. Major Time Effects (Exercise-induced changes)

Biomarker	Effect	p-value	Effect Size (ηp^2)	Interpretation
Protein	Time effect	<0.001***	0.823	Very large effect
Albumin	Time effect	<0.001***	0.756	Very large effect
Creatinine	Time effect	<0.001***	0.892	Very large effect
Uric Acid	Time effect	<0.001***	0.845	Very large effect
IL-1β	Time effect	0.005**	0.634	Large effect
TNF-α	Time effect	<0.001***	0.789	Very large effect
C5a	Time effect	0.004**	0.667	Large effect

Biomarker	Effect	p-value	Effect Size (ηp^2)	Interpretation
MCP-1	Time effect	<0.001***	0.812	Very large effect

****, $p<0.01$, and ***, $p<0.001$. Effect Size Interpretation (Cohen's guidelines): Small effect: $\eta p^2 = 0.01$-0.06, $d = 0.2$-0.5; Medium effect: $\eta p^2 = 0.06$-0.14, $d = 0.5$-0.8.**
